# Self-DNA release and STING-dependent sensing drives inflammation to cigarette smoke in mice

**DOI:** 10.1038/s41598-019-51427-y

**Published:** 2019-10-16

**Authors:** Mégane Nascimento, Aurélie Gombault, Norinne Lacerda-Queiroz, Corinne Panek, Florence Savigny, Malak Sbeity, Manon Bourinet, Marc Le Bert, Nicolas Riteau, Bernhard Ryffel, Valérie F. J. Quesniaux, Isabelle Couillin

**Affiliations:** University of Orleans and CNRS, INEM-UMR7355, Orleans, France

**Keywords:** Pattern recognition receptors, Chronic obstructive pulmonary disease

## Abstract

Cigarette smoke exposure is a leading cause of chronic obstructive pulmonary disease (COPD), a major health issue characterized by airway inflammation with fibrosis and emphysema. Here we demonstrate that acute exposure to cigarette smoke causes respiratory barrier damage with the release of self-dsDNA in mice. This triggers the DNA sensor cGAS (cyclic GMP-AMP synthase) and stimulator of interferon genes (STING), driving type I interferon (IFN I) dependent lung inflammation, which are attenuated in cGAS, STING or type I interferon receptor (IFNAR) deficient mice. Therefore, we demonstrate a critical role of self-dsDNA release and of the cGAS-STING-type I interferon pathway upon cigarette smoke-induced damage, which may lead to therapeutic targets in COPD.

## Introduction

Chronic obstructive pulmonary disease (COPD) is a severe chronic inflammatory disease associated with impaired lung functions. This disease is characterized by chronic lung inflammation which can lead to critical tissue destruction in some cases. The major cause of COPD is cigarette smoking. However other triggers such as air pollution are known contributors. Available treatments display only limited efficacy attempting to inhibit chronic inflammation characterized by the recruitment and activation of both innate (neutrophils and macrophages) and adaptive (T and B lymphocytes) immune cells in the small airways^1^. Immune and tissue cells, including epithelial and endothelial cells as well as fibroblasts, secrete a variety of proinflammatory mediators, in particular chemokines, cytokines and others mediators^2^.

Inflammation observed in COPD patients, smokers and sometimes even ex-smokers, is often driven by oxidative stress characterized by excessive reactive oxygen species production leading to DNA damage, cell death and subsequent pulmonary inflammation^1,3^. Indeed, cigarette smoke-induced cell death is characterized by danger associated molecular patterns (DAMPs) release contributing to persistent neutrophilic airway inflammation characteristic of COPD. Double-stranded (ds) DNA was shown to strongly correlate with neutrophilic inflammation^4^, causing inflammation and emphysema^5^. Nucleic acid sensing is a common and effective strategy to recognize microorganisms and initiate immune responses. However, it could also be an important feature of COPD^6^. Growing evidence demonstrates that pathogen receptors can also recognize self-nucleic acids in particular mitochondrial DNA (mtDNA) or nuclear DNA abnormally present in the cytosol^7,8^. Detection of these mislocalized self-nucleic acids can promote sterile inflammation and autoimmune diseases^9^. Among pattern recognition receptors (PRRs) involved in self-nucleic acid recognition, the dsDNA sensor TLR9 has been shown to participate to the development of emphysema caused by cigarette smoking in both mouse^10^ and human^11^. An increasing interest has emerged recently for stimulator of interferon genes (STING), an endoplasmic reticulum membrane-expressed protein. STING is activated by cyclic dinucleotides (CDNs), second messengers derived from microorganisms or synthesized by the enzyme cyclic GMP-AMP synthase (cGAS) after its binding to pathogen or self-derived dsDNA, including nuclear DNA and mtDNA^8,12–15^. DNA binding triggers cGAS to convert ATP and GTP into cGAMP, a CDN driving STING activation and cytokine production including type I interferons (type I IFN)^8,16,17^. STING is an adapter protein of nucleic acid sensors, located at the crossroads of several intracellular signaling pathways. In addition to cGAS, other cytosolic receptors (e.g. DDX41, IFI16) can sense DNA or CDNs and activate STING^18,19^. Its activation triggers transcription factors such as interferon regulatory factor 3 (IRF3) or nuclear factor κB (NF-κ B) and cytokine production including type I IFN involved in host immune responses. The type I IFNs (IFN I) family is a multi-gene cytokine family comprising 13 subtypes of the IFN-α family in human (14 in mouse) and a single IFN-β subtype^20^. While primarily involved in responses against viral and bacterial infections, the role of IFN I in other disease settings such as autoimmunity is well established^21,22^. IFNs I interact with the specific IFNAR membrane receptor, leading to the transcription of Interferons Stimulated Gene (ISG)^23^. More recently, it was shown that STING activation also leads to the secretion of type III IFNs^24,25^ and IL-1β^26^.

The immune consequences of self-nucleic acid release and detection by the cGAS/STING signaling pathway in pulmonary chronic inflammation and COPD is largely unknown. We showed previously that IL-1β and the inflammasome adaptor ASC are essential to cigarette smoke-induced inflammation, or elastase-induced emphysema in mice indicating that the inflammasome is involved in the establishment of COPD^27,28^. We identified uric acid as an endogenous DAMP released upon elastase-induced lung injury and activating the NLRP3/ASC inflammasome, driving IL-1β-dependent lung inflammation and emphysema^28^. Here we report a critical role of the cGAS/STING/type I interferon pathway in cigarette smoke-induced lung inflammation.

## Results

### Cigarette smokeexposure induces self-DNA release in the bronchoalveolar space

**Figure 1:**
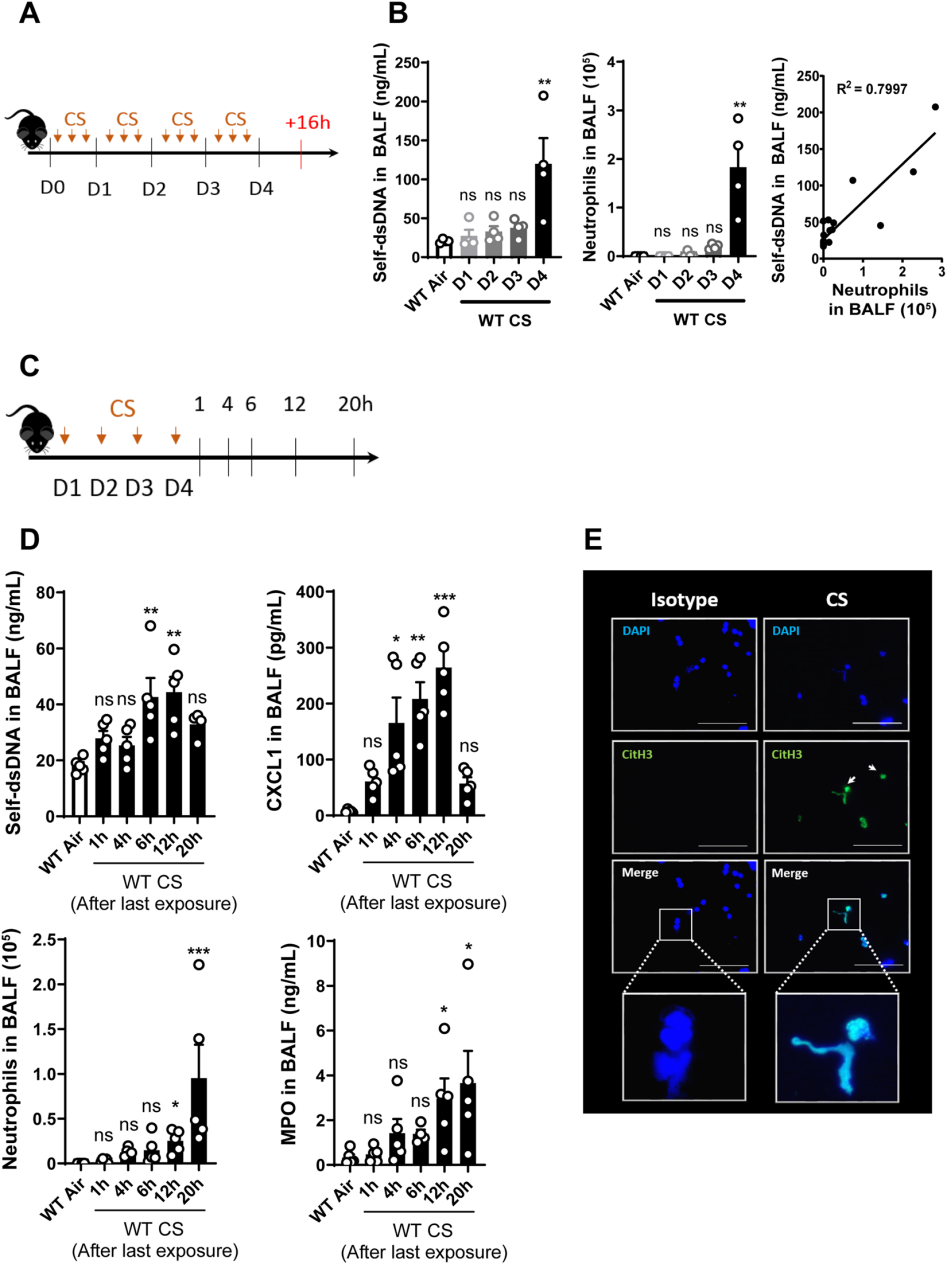
Acute cigarette smoke- (CS) exposure induces DNA release and neutrophil influx in BALF. Mice were exposed to CS three times a day and euthanized  16h after 1, 2, 3 or 4 days of exposure (**A**). Self-dsDNA content in BALF of WT mice exposed to CS after 1, 2, 3 or 4 days, neutrophil count and, correlation between self-dsDNA and neutrophils were shown. (**B**). Mice were exposed during 4 days and euthanized 1, 4, 6, 12 or 20 h after the last exposure (**C**). Self-dsDNA and CXCL1 levels, neutrophils and MPO in BALF were shown (**D**). Immunostaining of citrullinated histone H3 (CitH3) in green and DNA in blue was performed on BAL cells of WT-CS mice at day 4, 16 h after the last exposure. Scale bars = 100 µm (**E**). Bar graph are expressed ± SEM.

Cigarette smoke (CS)-exposure was shown to induce cell damage and death with free DNA release^4^ and we first analyzed the DNA-content in the airways of acutely CS-exposed mice. C57BL/6 wild-type (WT) mice were exposed three times a day and euthanized 16 h after 1, 2, 3 or 4 days of exposure (Fig. 1A). Self-dsDNA increase in bronchoalveolar lavage fluid (BALF) was detectable only on day 4, together with neutrophil recruitment in the airways (Fig. 1B). Importantly, self-dsDNA level and neutrophil influx into the BALF are correlated suggesting that CS-induced self-DNA may act as a proinflammatory signal for neutrophil recruitment (Fig. 1B). A kinetic analysis of DNA release and inflammation was performed at 1, 4, 6, 12 or 20 h after the last exposure of a 4 days CS-exposure protocol (Fig. 1C). The data show that self-dsDNA is detectable at 6 h after the last exposure on day 4, preceding neutrophil recruitment which is visible between 12 h and 20 h (Fig. 1D). The neutrophil attracting chemokine CXCL1 (C-X-C motif chemokine)/KC is increased in BALF between 4 h to 12 h after the last CS-exposure (Fig. 1D). In addition, neutrophil influx into the BALF is confirmed by the increased level of myeloperoxidase (MPO) in the BALF at 12 and 20 h after the last CS-exposure, thus after CXCL1/KC production (Fig. 1D). These results indicate that 4 days CS-exposure induce self-dsDNA release, with kinetics preceding neutrophil recruitment into the broncho-alveolar space. Nevertheless, high self-dsDNA levels persist 20 h after the last exposure at time where BALF neutrophil influx was the most important, suggesting that neutrophils may also release their own DNA as neutrophil extracellular traps (NETs). To evaluate NET formation in response to CS-exposure, we performed immunofluorescence staining of bronchoalveolar lavage (BAL) cells collected from CS-exposed WT mice showed a specific signal for citrullinated histone H3, a specific marker of NETs. This signal colocalized with DAPI, suggesting that neutrophils recruited upon CS-exposure also release their DNA as citrullinated histone H3-containing NETs (Fig. 1E). In conclusion, CS-induced self-DNA release from damaged lung cells, promotes the recruitment of neutrophils that amplify airway inflammation through NET-mediated DNA release.

### Acute CS-exposure increases cGAS and STING expression in the lung

**Figure 2:**
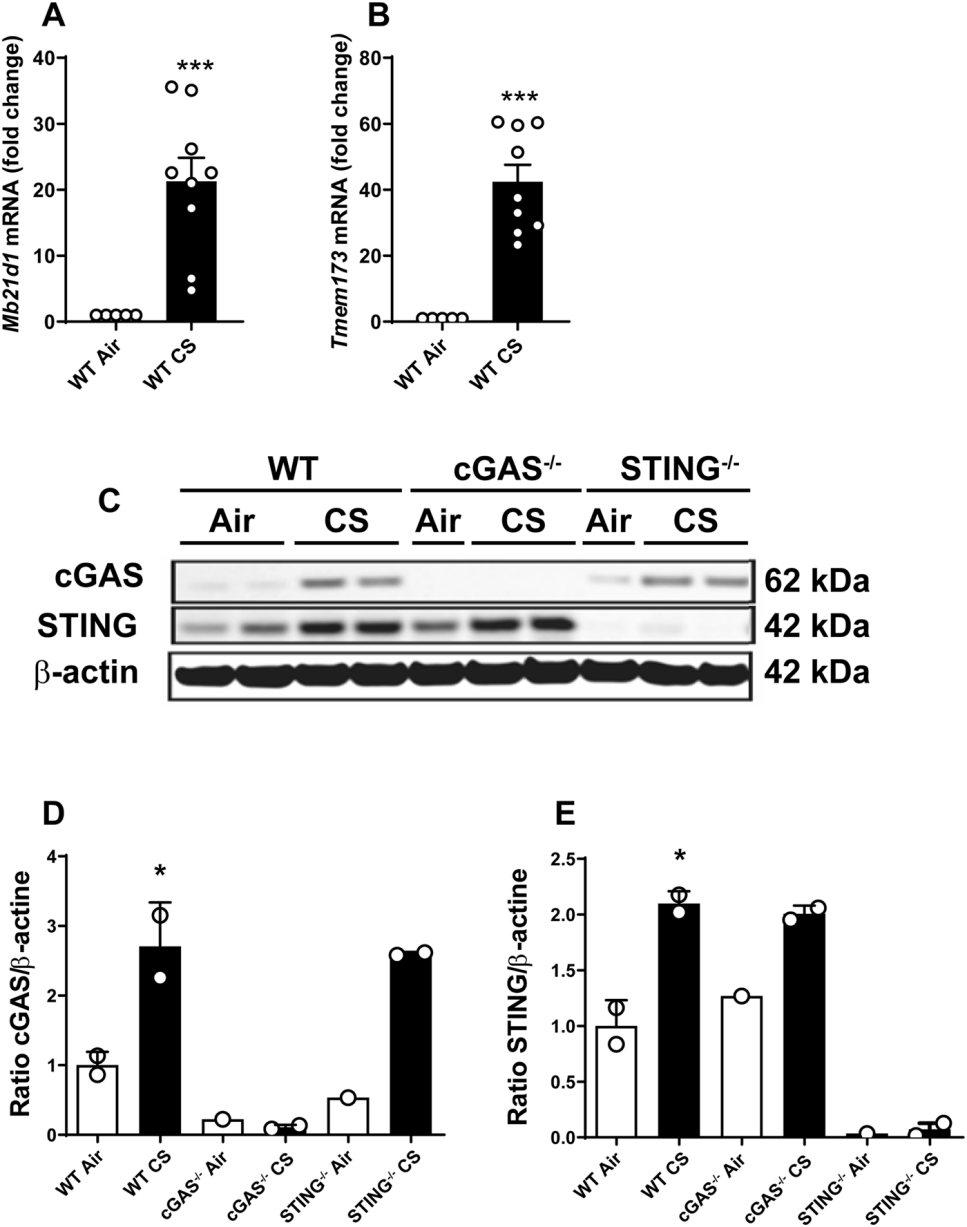
Acute CS-exposure induces cGAS and STING expression. *Mb21d1* (cGAS) (**A**), *Tmem173* (STING) (**B**) mRNA expression in lung homogenates in Air- and CS-exposed mice were shown. Immunoblot for cGAS and STING proteins under reducing conditions in lung homogenates of Air and CS mice with beta actin as reference (**C**) and quantification of cGAS (**D**) and STING (**E**) immunoblot were shown. Bar graph are expressed ± SEM.

We then asked whether the DNA sensor cGAS and the adaptor protein STING are involved in the sensing of self-dsDNA released upon CS-exposure, and analysed their expression in acutely CS-exposed mice. CS-exposed WT mice overexpressed  cGAS (*Mb21d1*) (Fig. 2A), and STING (*Tmem173*) (Fig. 2B) mRNA in the lungs at 4 days of CS-exposure. Furthermore, CS-exposure increased pulmonary expression of the cGAS and STING proteins in WT mice over control air-exposed mice while STING^−/−^ mice or cGAS^−/−^ mice did not express cGAS or STING, as expected (Fig. 2C–E; Supplementary Fig. S2). Our data indicate that self-dsDNA released is accompanied by the overexpression of the DNA sensor cGAS and the adaptor protein STING after acute CS-exposure. These results suggest that early dsDNA release induces cGAS and STING expression which is in line with an engagement of the cGAS/STING pathway in pulmonary inflammatory response to CS-exposure.

### Cigarette smoke-induced lung inflammation is STING dependent

**Figure 3:**
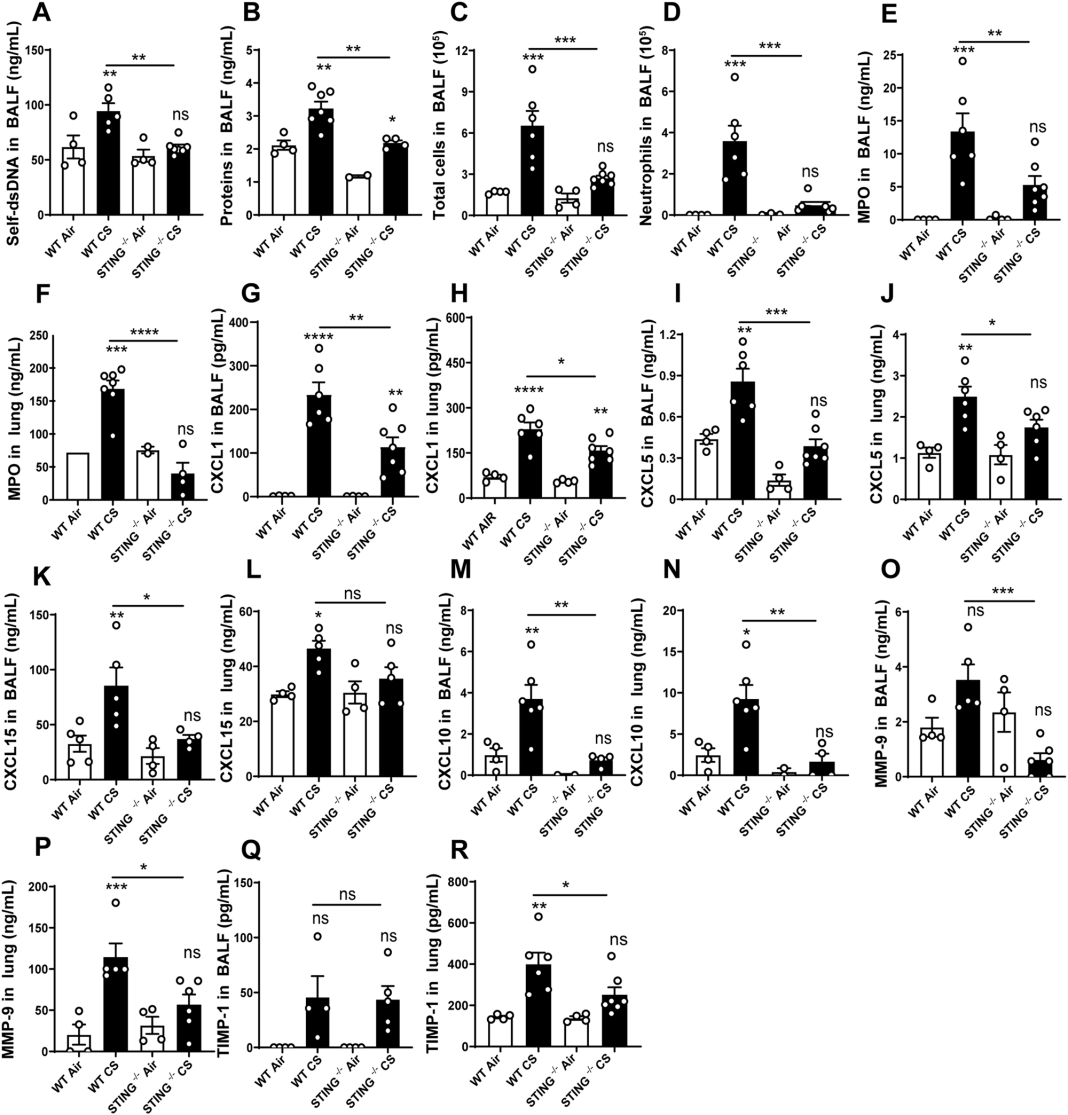
Cell recruitment induced by CS-exposure is decreased in STING^−/−^ mice. Self-dsDNA (**A**) and protein levels (**B**) were measured in BALF. Total cells (**C**), neutrophils (**D**), MPO level in BALF (**E**) and lung (**F**) are shown. The level of CXCL1 (**G**,**H**), CXCL5 (**I**,**J)**, CXCL15 (**K**,**L)** and CXCL10 (**M**,**N)** respectively in BALF and lung are shown. Remodeling factors MMP-9 (**O,P**) and TIMP-1 (**Q,R**) were measured respectively in BALF and lung are shown. Bar graph are expressed ± SEM.

Since STING is overexpressed in the lung of CS-exposed mice, we next investigated whether the STING pathway is required for CS-induced lung inflammation. We exposed wild-type or STING deficient (STING^−/−^) mice to CS for 4 days and analysed the pulmonary inflammation. The increase of dsDNA levels in BALF observed in CS-exposed WT mice was significantly reduced in the BALF of CS-exposed STING^−/−^ mice (Fig. 3A), suggesting that self-dsDNA is released *de novo* dependent on STING. In addition, CS-exposure induced an increase in protein extravasation in the BALF of WT mice, but not in STING^−/−^ mice indicating a reduced respiratory barrier damage in the absence of STING (Fig. 3B). Total inflammatory cell and neutrophil counts recovered in the BALF were decreased in STING^−/−^ CS mice as compared to WT CS mice (Fig. 3C,D). Among immune cells, neutrophils are known to play a major role in response to CS^29,30^. As a correlate of neutrophil recruitment, the neutrophil marker MPO was significantly reduced in the BALF and lungs of CS-exposed STING^−/−^ mice as compared to WT mice (Fig. 3E,F). Analyzing the expression of the neutrophil attracting chemokines, we observed that BALF and lung levels of CXCL1/KC (Fig. 3G,H), CXCL5/LIX (Fig. 3I,J) and CXCL15/Lungkine (Fig. 3K,L) were significantly lower after CS-exposure in STING^−/−^ mice as compared to WT mice. In addition, BALF and lung levels of the IFN I downstream CXCL10/IP-10 chemokine were not increased in STING^−/−^ CS mice after exposure as compared to CS-exposed WT mice (Fig. 3M,N). Finally, levels of the remodeling factors matrix metalloproteinase (MMP)-9 (Fig. 3O,P) and tissue inhibitor of metalloproteinases (TIMP)-1 (Fig. 3Q,R) in lungs were reduced in CS-exposed STING^−/−^ mice in comparison to CS-exposed WT mice. Altogether these results indicate that the STING signaling cytosolic protein is a key player in pulmonary inflammatory responses to CS-exposure.

### DNA sensor cGAS, but not TLR9, is required for CS-induced lung inflammation

**Figure 4:**
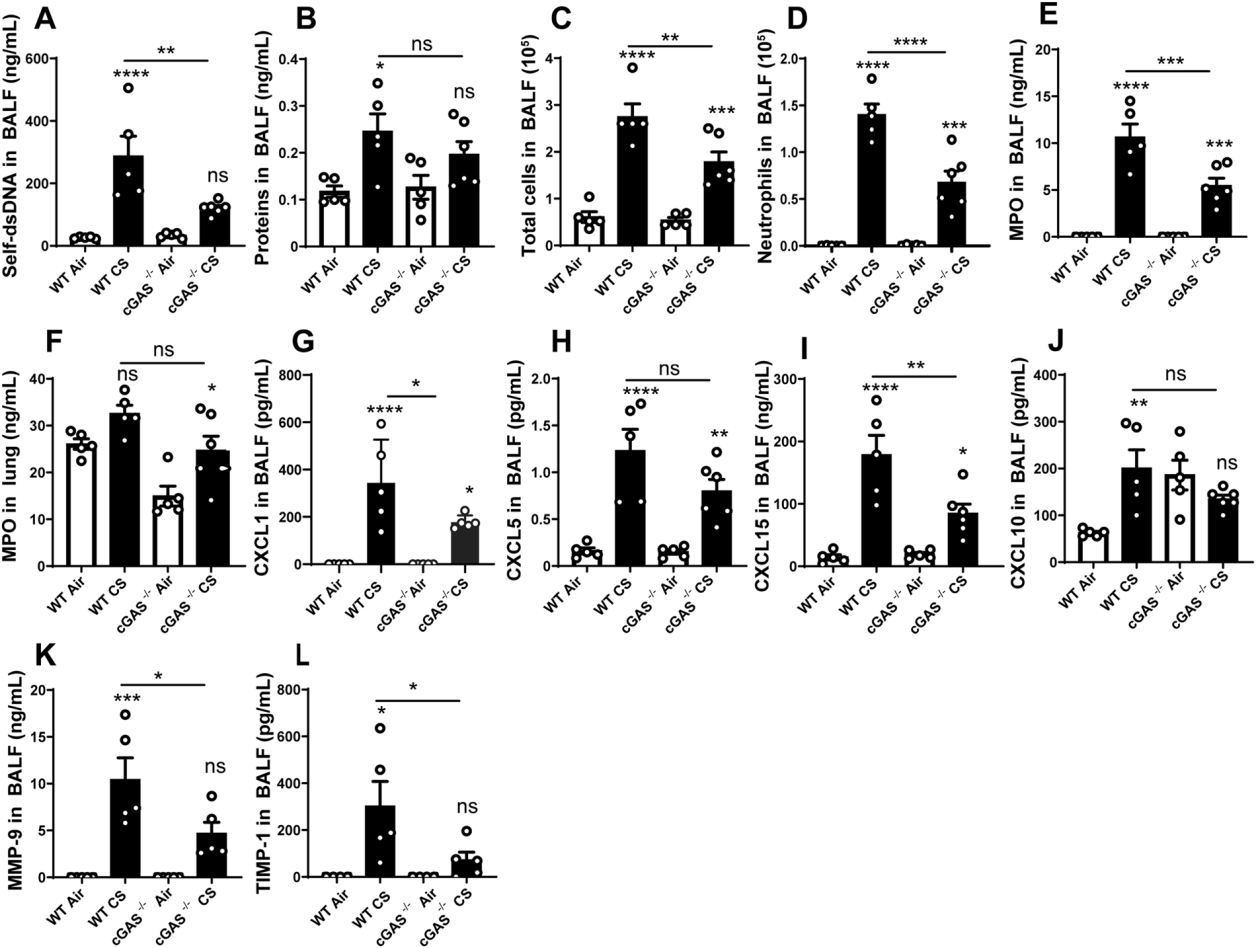
Cell recruitment induced by CS-exposure is decreased in cGAS^−/−^ mice. Self-dsDNA (**A**) and protein levels (**B**) were measured in BALF. Total cells (**C**), neutrophils (**D**) and, MPO levels in BALF (**E**) and lung (**F**) are shown. The level of CXCL1 (**G**), CXCL5 (**H**), CXCL15 (**I**), CXCL10 (**J**), MMP-9 (**K**) and TIMP-1 (**L**) in BALF are shown. Bar graph are expressed ± SEM.

To investigate whether the cGAS sensor is involved in CS-induced DNA sensing and lung inflammation, we exposed cGAS deficient mice (cGAS^−/−^) to CS for 4 days. Compared to WT mice, cGAS^−/−^ CS-exposed mice presented less self-dsDNA in BALF (Fig. 4A) and a slight reduction in protein extravasation in BALF which did not reach statistical significance (Fig. 4B), suggesting a cGAS-dependent barrier injury. In addition, cGAS^−/−^ mice exposed to CS presented a reduced recruitment of total cells, neutrophils (Fig. 4C,D) and MPO levels in BALF and lung (Fig. 4E,F) as compared to CS-exposed WT mice. Moreover, there was some reduction in CXCL1/KC, CXCL5/LIX, CXCL15/Lungkine and CXCL10/IP-10 in the BALF of CS-exposed cGAS^−/−^ mice (Fig. 4G–J). The levels of remodeling factors MMP-9 and TIMP-1 were reduced in the BALF of CS-exposed cGAS^−/−^ mice as compared to WT mice (Fig. 4K,L). Since the expression of TLR9, another self-dsDNA sensor, has been reported in CS-induced emphysema in mice^10^ and in humans^11^, we also exposed TLR9 deficient mice (TLR9^−/−^) to CS during 4 days and analysed the inflammatory response. CS-exposed TLR9^−/−^ mice exhibited similar self-DNA levels (Supplementary Fig. S1A), total cells, macrophages and neutrophils in the BALF (Supplementary Fig. S1B–D), as compared to CS-exposed WT mice, together with similar MPO, CXCL1 and CXCL5 levels in the BALF (Supplementary Fig. S1E–G), indicating that self-dsDNA release and pulmonary inflammation to acute 4 days CS-exposure are independent of TLR9 signaling. All together these results indicate that cGAS but not TLR9, is involved in self-dsDNA sensing and lung inflammation after CS-exposure, likely through the STING signaling pathway.

### Cigarette smoke-exposure induced pulmonary inflammation is mediated by type I interferons

**Figure 5:**
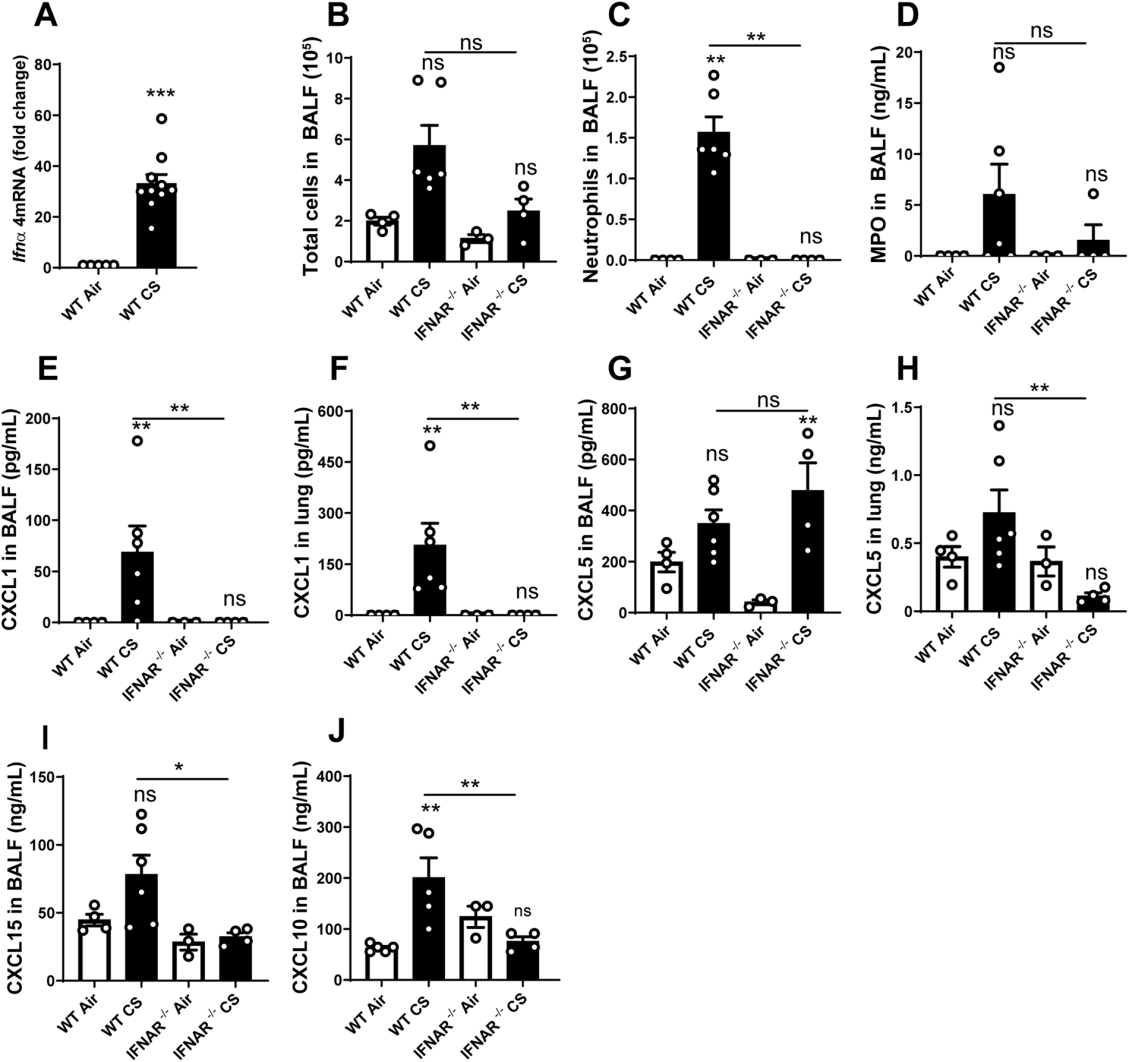
Type I interferon response is involved in CS-induced lung inflammation. *Ifnα4* mRNA expression (**A**) in lung of Air or CS-exposed mice, total cells (**B**), neutrophils (**C**) and MPO level (**D)** in BALF after CS-exposure are shown in IFNAR^−/−^ mice. CXCL1 (**E**,**F**) and CXCL5 (**G**,**H**) chemokines were measured in BALF and lung. CXCL15 (**I**) and CXCL10 (**J**) measured in BALF are shown. Bar graph are expressed ± SEM.

We next investigated the role of type I IFNs in CS-induced lung inflammation. Type I IFNs expression was increased in the lung of WT mice exposed to CS as measured by the overexpression of *Ifnα*4 mRNA (Fig. 5A). We exposed WT mice and mice deficient for the type I IFN receptor (IFNAR^−/−^) to CS. IFNAR^−/−^ mice presented attenuated total cell (Fig. 5B), neutrophil (Fig. 5C) recruitment and MPO levels (Fig. 5D) in the BALF. There was a great reduction of CXCL1/KC in the BALF or lung of CS-exposed IFNAR^−/−^ mice (Fig. 5E,F) as well as the chemokine CXCL5 (Fig. 5G,H). CS-exposed IFNAR^−/−^ mice also produced reduced levels of CXCL15/Lungkine in the BALF (Fig. 5I) as compared to CS-exposed WT mice. In addition, BALF levels of the downstream CXCL10 chemokine were reduced in IFNAR^−/−^ mice (Fig. 5J). Altogether, these results demonstrate that type I IFN signaling through the IFNAR pathway plays a critical role in the inflammatory response to CS exposure in mice through cGAS/STING signaling.

## Discussion

We show that mouse CS-exposure promotes self-dsDNA release which correlates with neutrophil influx into the broncho-alveolar space. dsDNA may act as a proinflammatory signal for pulmonary inflammation^4^. Self-dsDNA may originate from damaged cells such as alveolar epithelial cells and macrophages, but also from neutrophilic death, possibly through NETosis which was shown to be an integral part of CS-induced experimental inflammation and human COPD, amplifying airway inflammation^31,32^. Importantly, defective repair of DNA damage was implicated in the pathogenesis of COPD, suggesting a key role for DNA release and sensing^33,34^. In addition, incomplete tobacco combustion generates carbon black nanoparticles (nCB). Nanoparticles accumulation in pulmonary dendritic cells and macrophages initiates and sustains lung inflammation, promoting emphysema development^35^. In mice and COPD patients, nCB uptake by macrophages induces DNA repair enzymes leading to dsDNA breaks^5^. The nucleus or the mitochondria can be a source of self-dsDNA triggering DNA sensors activation after CS-exposure, as shown for silica particles^36^.

Importantly, we report that CS-exposure triggers cGAS and STING expression at both mRNA and protein levels. In addition, cGAS/STING pathway is involved in pulmonary inflammation and remodeling upon CSexposure. Furthermore, part of self-dsDNA release is dependent on cGAS and STING indicating that pulmonary damage induced *de novo* cell death and self-dsDNA dependent lung inflammation through an amplification loop^37^. We performed an immunostaining of BAL cells using a specific citrullinated histone H3 antibody. We observed that cells from CS-exposed mice displayed a citrullinated histone H3 signal that co-localizes with specific DNA marker DAPI indicating the presence of NETs. These results suggest that self-DNA measured in the BALF may also originate from dying recruited neutrophils through NET formation. In addition, using gene deficient mouse strains we show that the absence of cGAS or STING leads to attenuated lung inflammation including reduction of type I IFN/IFNAR-dependent secretion of the interferon inducible gene (ISG) CXCL10. Even if other pathways may lead to type I IFN production, STING activation is widely recognized in the literature as a type I IFN inducer. Therefore, our results support the concept that the cGAS/STING/type I interferon pathway plays an important role in CS-induced pulmonary inflammation even if other pathways exist.

We identify cGAS as a critical cytosolic DNA sensor upstream of STING implicated in lung inflammation to CS, whereas TLR9 receptor, another DNA sensor protein, is dispensable. Other molecules contributing to cytosolic DNA sensing and type I IFN pathway activation in a STING-dependent manner may also be involved in particular DDX41, IFI16 or mouse IFI204, DAI or others^38–43^. In addition, other danger signal receptors independently of STING or TLR9 may be activated leading to type I IFN/IFNAR-dependent pulmonary inflammation including MDA5/RIG-l, TLR4/MyD88, TLR7MyD88 or TLR8/MyD88,). Indeed, during CS-induced cell death or stress, several signaling pathways may be activated, converging in type I interferon secretion. However, our results showing a clear decrease in CXCL1, neutrophil influx and inflammation in cGAS and STING deficient mice demonstrate that cGAS/STING pathway participates in the establishment of lung inflammation upon CSexposure. Open questions remain and include how dsDNA released upon injury is transported into the cytosol to interact with cGAS.

Our study emphasizes that viruses and inorganic particles share similarities in regards to the signaling pathways involved and especially here the cGAS/STING/IFNAR pathway. In line with this statement, it was shown that CS increases poly (I:C)-induced airway inflammation, possibly via an increased expression of the poly (I:C) sensor TLR3^44^. However, others studies indicated that intratracheal CS extract administration or whole body CS exposure can cause antiviral immunosuppression in these mice, inhibiting RIG-I induction as well as IFN-β and CXCL10 expression *in vivo*^45^. Importantly, CXCL10 was shown to control the secretion of elastolytic MMPs in lung tissue macrophages in former smokers with emphysema devoid of infection, suggesting autoinflammatory mechanisms in COPD^35,46^.

We showed previously that type I IFN production with IFNAR signaling^47^ and more recently self-DNA release and cGAS/STING-dependent type I IFN secretion^36^ drive silica-induced lung inflammation confirming the important role of this pathway in particle-induced lung inflammation and pathology. We demonstrate here that self-dsDNA sensing and cGAS/STING pathway are crucial in CS-driven lung inflammation. We reported that CS- and silica-induced inflammation depend on the NLRP3 inflammasome^27,28^ and showed that STING may also participate to NLRP3 inflammasome activation and induced pulmonary inflammation^36^.

Interestingly, a recent study showed that in human myeloid cells cytosolic DNA sensing by cGAS/STING triggers potassium efflux that activates NLRP3 inflammasome, leading to IL-1β secretion independently of type I IFN secretion^48^. These results illustrate the complexity of innate immune regulation to cell death and damage.

In conclusion, we show here that cigarette smokeexposure causes cell injury with self-dsDNA release triggering cGAS/STING pathway and leading to type I IFN secretion and pulmonary inflammation. Thus, STING might represent a potential therapeutic target in order to control lung inflammation upon cigarette smoking and prevent COPD development.

## Materials and Methods

### Mice

Wild-type C57BL/6J (WT) male mice were purchased from the Janvier laboratory (Janvier Laboratory, France). IFNAR^−/−^ were provided by Michel Aguet from the Institute of Molecular Biology I, University of Zurich, Switzerland^49^, STING^−/−^ by Glen Barber^50^, cGAS^−/−^ mice by Zhijian Chen^51^ and TLR9^−/−^ by Shizuo Akira^52^. All mice were backcrossed 10 times or made on C57BL/6 J background and housed in the UPS44-TAAM (CNRS, Orleans, France) animal facility. For experiments, adults (8–12 weeks old) were kept in sterile, isolated and ventilated cages. All animal experiments followed the French government’s ethical and animal experiment regulations and were approved by the “Ethics Committee for Animal Experimentation of CNRS Campus Orleans” (CCO) under number CLE CCO 2015-1088.

### Cigarette smoke model

3R4F cigarettes (University of Kentucky, Lexington, KY) were used without filter. Mice were placed in a smoke chamber of InExpose system (EMKA Technologies, Paris, France) and exposed to the smoke of four cigarettes per exposure, three times a day, for four consecutive days. Bronchoalveolar lavage (BAL) and lung tissue were harvested 16 hours after the last exposure.

### Bronchoalveolar lavage (BAL)

BAL was performed as previously described^27^. Differential cell counts were performed by counting an average of 250 cells on Cytospin preparations (Shandon CytoSpin3, Thermo Scientific, Illkirch, France) after May-Grünwald-Giemsa (MGG) staining (Diff Quick, Medion Diagnostics, Düdingen, Switzerland) according to manufacturer’s instructions.

### Lung homogenates

After BAL collection, lungs were perfused with Isoton® (Beckman Coulter France, Villepinte) to flush the vascular content. Lungs were homogenized by a rotor-stator (Ultra-turrax®) in 1 ml of PBS for ELISA dosage or in RIPA buffer (Cell Signaling Technology, Leiden, The Netherlands) containing anti-proteases/anti-phosphatases cocktail (ThermoFisher Scientific, Waltham, MA) for immunoblotting. The extract was centrifuged 10 min 10000 rpm and the supernatant was stored at −80 °C before mediator measurement and immunoblotting analysis.

### Measurement of double-stranded DNA

Double-stranded DNA was measured in the BAL fluid (BALF) using Quant-iTPicoGreen dsDNA reagent (Invitrogen, Carlsbad, CA), according to the manufacturer’s protocol.

### Mediator measurements

For cytokine determination, BALF supernatants and lung homogenates were analysed by ELISA assay kits for murine: CXCL1, CXCL5, CXCL10, CXCL15, MPO, MMP-9 and TIMP-1 (R&D system, Minneapolis, USA) according to manufacturer’s instructions. Protein levels in BALF were measured by Pierce™ BCA Protein Assay Kit (ThermoFisher Scientific, 23225).

### Quantitative RT-PCR

RNA was purified from lung homogenates by using Tri-Reagent (Sigma-Aldrich, Saint-Louis, MO) extraction protocol. Reverse transcription of RNA into cDNA was carried out with GoTaq qPCR-Master Mix (Promega, Madison, WI). RT-qPCR was performed with Fast SYBR Green Master mix (Promega) on an ARIA MX (Agilent Technologies, Santa Clara, CA). Primers for *Tmem173 (*#QT00261590)*, Mb21d1 (*#QT00131929) *and Ifnα4 (*#QT01774353) were purchased from Qiagen (Qiagen, Hilden, Germany). RNA expression was normalized to *Gapdh* (#QT00166768) expression and analysed using the ^ΔΔ^Ct method.

### Immunoblotting

Protein concentrations were determined in lung tissue by using Pierce BCA protein assay (ThermoFisher Scientific). 40 µg of proteins were denatured by boiling (95 °C, 5 min) in reducing SDS sample buffer, separated by SDS-PAGE and transferred to nitrocellulose membranes (GE Healthcare Life Sciences, Amersham, UK). The membranes were blocked 2 hours in 5% Blotting-Grade Blocker (BioRad, France), washed three time in Tris-Buffered saline (TBS)- 0,1% Tween® 20 and incubated with primary mouse anti-STING (Abcam, ab92605) and anti-cGAS (Cell Signaling Technology, #3165) antibody in TBS-BSA (Bovine Serum Albumine) 1%- azide 0,5 mM overnight at 4 °C. Membranes were then washed three times in TBS-0,1% Tween® 20 and incubated with the appropriate second antibody conjugated to horseradish peroxidase (HRP) two hours at Room Temperature (RT). Membranes were incubated with mouse anti-actin HRP-conjugate (Sigma-Aldrich) in 5% Blotting-Grade Blocker in TBS-0,1% Tween® 20 for 2 hours at RT. Detection was performed with ECL Western-blotting Detection Reagent (GE Healthcare). The intensity of bands revealed was quantified by mean of densitometry with Multi-application gel imaging system PXi software (Syngene).

### Immunostaining on Cytospin

Cytospin preparations without MGG staining were fixed in paraformaldehyde 4% (Sigma-Aldrich). After 3 lavages in TBS, Cytospins were incubated 10 min in TBS-0.3% Triton X-100. Then, Cytospins were washed 3 times in TBS, blocked in TBS-1% BSA-10% SVF during 45 min and incubated with primary anti-histone H3-citrulline (Abcam, ab5103) or control isotype antibodies over night at 4 °C. After 3 lavages in TBS, Cytospins were incubated 1 hour at room temperature with anti- Rabbit IgG conjugated to Alexa 488. Cytospins were counterstained using 4′,6-diamidino-2-phenylindole (DAPI) for 10 min, rinsed and coverslip were mount with Mowiol (Sigma-Aldrich). Cells were observed using a Nikon eclipse 80i microscope and images were treated using ImageJ software.

### Statistical analysis

Statistical evaluation of differences between experimental groups was determined by Mann-Whitney or one-way ANOVA, analysis of variance, Bonferroni test for *in vivo* experiments using Prism software (La Jolla, CA, USA). *P* values < 0.05 were considered statistically significant. *p < 0.05, **p < 0.01, ***p < 0.001, and ns : non significant.

## Supplementary information

Supplementary Figures
