# Supplementary material for: Self-DNA release and STING-dependent sensing drives inflammation to cigarette smoke in mice
Source: Sci Rep. 2019 Oct 16;9:14848. doi: 10.1038/s41598-019-51427-y (PMC6795997; doi:10.1038/s41598-019-51427-y)
Supplement: Supplementary file 1 — Supplementary Figures [file 41598_2019_51427_MOESM1_ESM.pdf]

Self-DNA release and STING-dependent sensing drives inflammation to cigarette smoke in mice

Mégane Nascimento[1], Aurélie Gombault[1], Norinne Lacerda-Queiroz[1], Corinne Panek[1], Florence Savigny[1], Malak Sbeity[1], Manon Bourinet[1], Marc Le Bert[1], Nicolas Riteau[1], Bernhard Ryffel[1], Valérie F.J. Quesniaux[1] & Isabelle Couillin[1]

[1]University of Orleans and CNRS, INEM-UMR7355, Orleans, France

Supplementary Figure S1

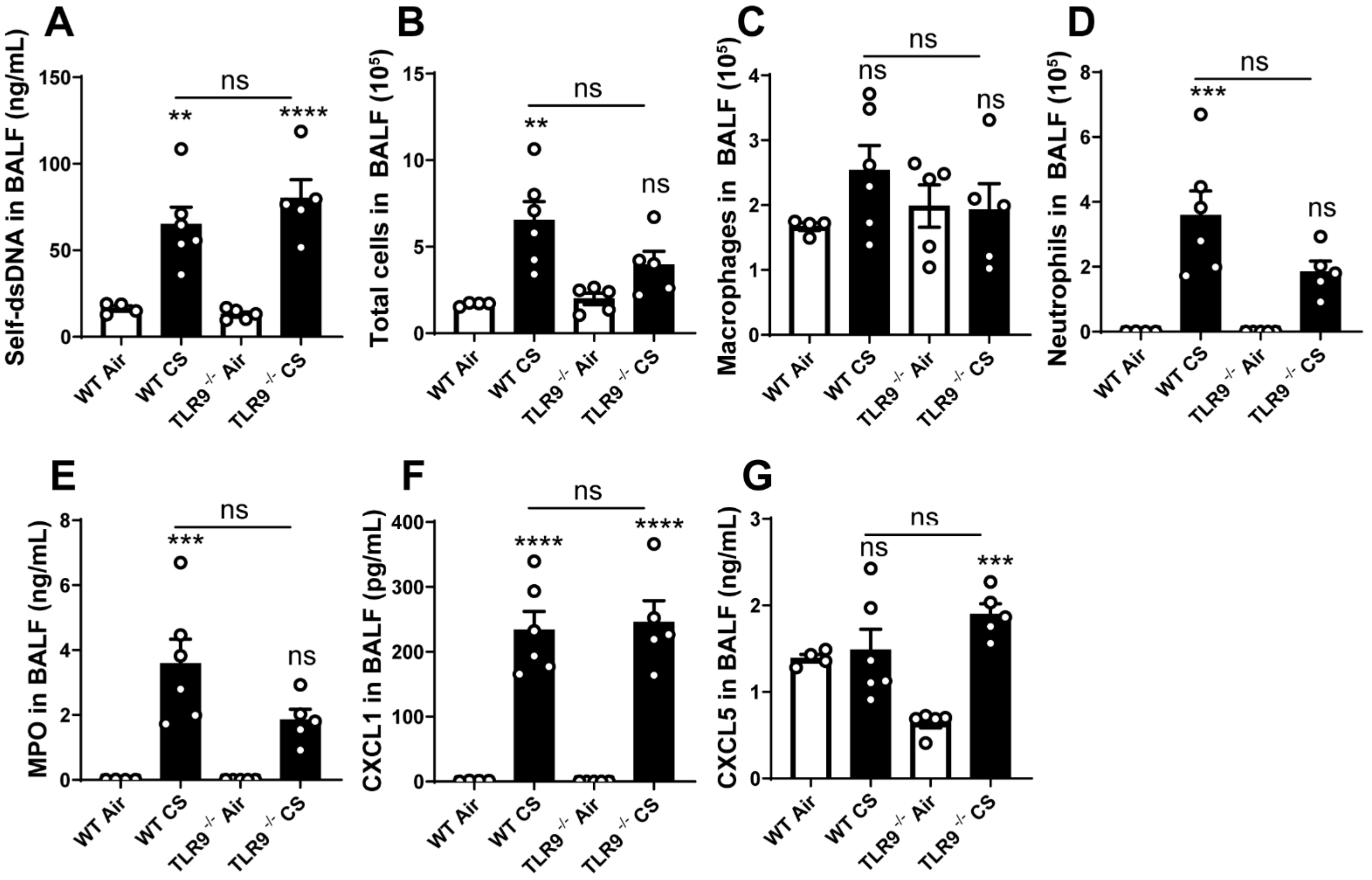

Supplementary Figure S2

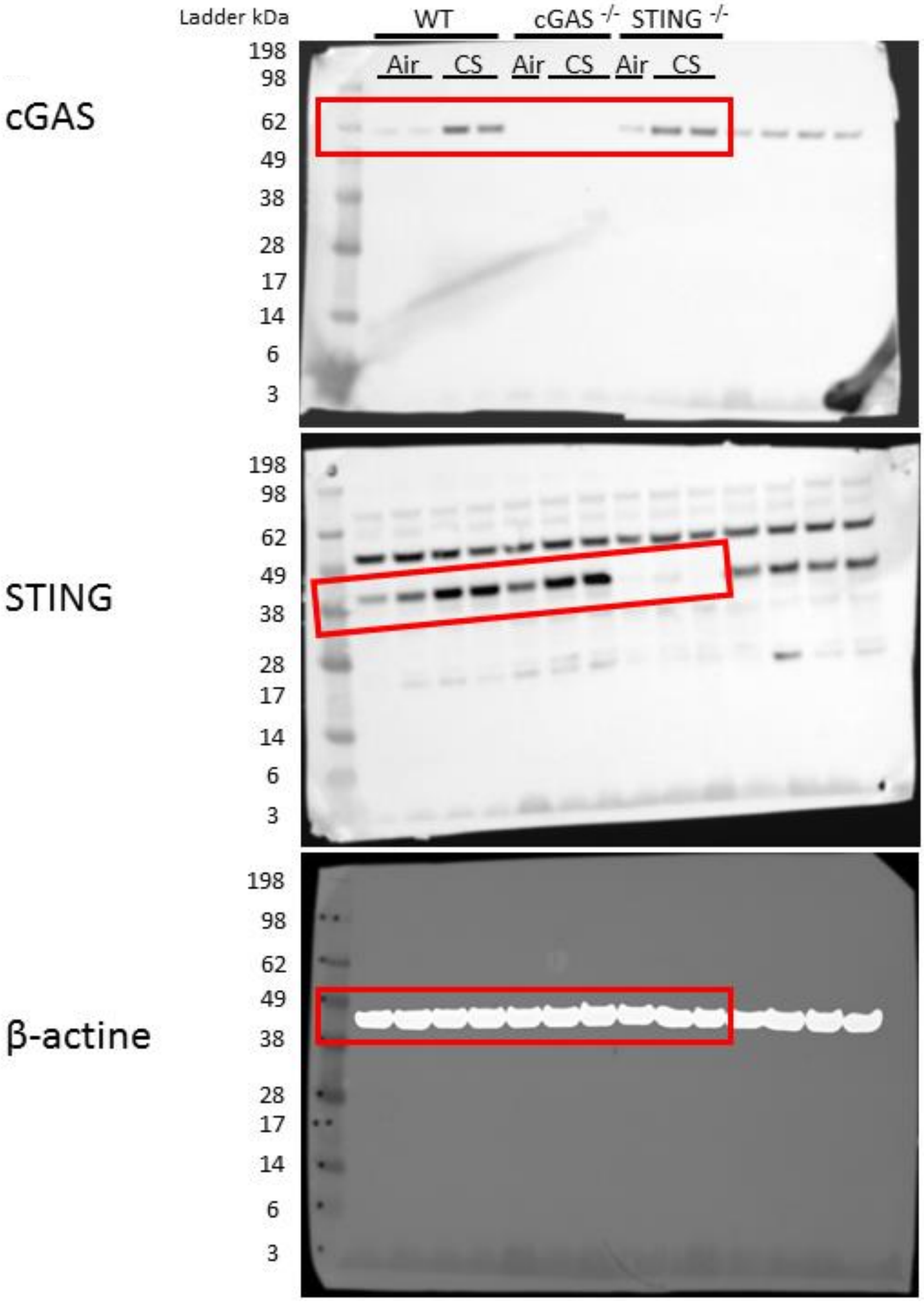

Supplementary Figure Legends

Supplementary Figure S1 DNA sensor TLR9 is not required in CS-induced lung inflammation. Self-dsDNA (**A**), total cells (**B**), macrophages (**C**) and neutrophils (**D**) recruited in BALF after CS exposure are shown. MPO (**E**), CXCL1 (**F**) and CXCL5 (**G**) levels measured in BALF are shown.

Supplementary Figure S2 Uncropped immunoblots shown in Fig.2C.
